# Supplementary material for: MetaRibo-Seq measures translation in microbiomes
Source: Nat Commun. 2020 Jun 29;11:3268. doi: 10.1038/s41467-020-17081-z (PMC7324362; doi:10.1038/s41467-020-17081-z)
Supplement: Supplementary file 10 — Supplementary Data 7 [file 41467_2020_17081_MOESM10_ESM.zip › File2/Confidence_High_Taxonomy/388638_out.krona.html]

Javascript must be enabled to view this page.

members
magnitude
magnitudeUnassigned
count
unassigned
taxon
rank

388638\_out

5

5
2
superkingdom

phylum
1239
5

91061
2
class

2
186826
order

2
81852
family

1350
2
genus


SRS015960\_contig\_number\_7572SRS064276\_contig\_number\_39628
species
2
1352

3
186801
class

186802
3
order

family
1
216572

1
1952970

SRS016203\_contig\_number\_contig-100\_13.78718
species

1897045
1

SRS056259\_contig\_number\_31339
species

1
31979
family

1485
1
genus

species

SRS104400\_contig\_number\_19818
59620
1
